# Fabrication of multifunctional alginate microspheres containing hydroxyapatite powder for simultaneous cell and drug delivery

**DOI:** 10.3389/fbioe.2022.827626

**Published:** 2022-08-09

**Authors:** Jueun Kim, Yeong-Jin Choi, Honghyun Park, Hui-suk Yun

**Affiliations:** ^1^ Department of Advanced Materials Engineering, University of Science & Technology (UST), Daejeon, South Korea; ^2^ Ceramic Materials Division, Department of Advanced Biomaterials Research, Korea Institute of Materials Science (KIMS), Changwon, South Korea

**Keywords:** alginate-hydroxyapatite, microsphere, cell and drug delivery, encapsulator, 3D ultrasonic

## Abstract

Novel alginate-hydroxyapatite hybrid microspheres were developed for simultaneous delivery of drugs and cells as a multifunctional bone substitute for osteoporotic bone tissue regeneration. The microspheres were used to enhance osteogenesis and to carry and deliver quercetin, a representative phytoestrogen that controls bone tissue regeneration metabolism in osteoporosis patients, through sustained release over a long period. To overcome quercetin’s hydrophobicity and low solubility in aqueous environments, we added it to the surface of hydroxyapatite (HAp) nanoparticles before mixing them with an alginate solution. The homogeneous distribution of the HAp nanoparticles in the alginate solution was essential for preventing nozzle clogging and achieving successfully fabricated hybrid microspheres. To this end, a 3D ultrasonic treatment was applied. Electrostatic microencapsulation was then used to fabricate hybrid alginate-HAp microspheres containing quercetin and cells. The microspheres were approximately 290.7 ± 42.5 μm (aspect ratio of 1). The sustained release of quercetin was confirmed during a test period of 20 weeks. The cells in the hybrid microspheres maintained good cell viability during the entire testing period, and their osteogenic differentiation behavior was boosted by the presence of HAp. Thus, osteogenic differentiation could be greatly improved by adding quercetin. These novel multi-biofunctional hybrid microspheres have great potential for the regeneration of osteoporotic bone tissue at indeterminate defect sites.

## Introduction

Osteoporosis is a common disease of the elderly, occurring regardless of gender. It is a systemic skeletal disease defined by low bone mass caused by bone metabolic degradation, which results from the bone resorption rate being higher than the bone formation rate (Dreifke et al., 2013; Compston et al., 2019). A common consequence is bone injury. Repairing fractures often involves bone grafts and bone cement, which is based on osteoconductive ceramics, such as calcium phosphate (CaP). It is also essential to reduce the mortality and other complications that may occur after fracture (Dimitriou et al., 2011; Wei et al., 2016). There is some risk of further and larger fractures in osteoporotic areas due to the relatively weakened bone. (Dennison et al., 2019). Therefore, an effective sustainable treatment is required, such as delivery of a drug or biomolecule.

Scaffolds are often used as drug or biomolecule carriers due to their ability to release drugs in an efficient and sustained manner over a long period. In general, two groups of scaffolds are used: implantable scaffolds (e.g., porous matrices and nanofiber mesh structures) and injectable scaffolds (e.g., hydrogels and microspheres) (Garg et al., 2012). Microspheres, in particular, have been shown to be excellent controlled-release vehicles and are used in injectable formulations in clinical applications, such as minimally invasive surgeries. Their physicochemical characteristics and versatility, which allow control of the release kinetics of encapsulated factors, make them ideal for use as carriers (Dhandayuthapani et al., 2011; Garg et al., 2012; Huang et al., 2014; Gupta V. et al., 2017).

Microspheres are conventionally fabricated *via* the water-in-oil emulsion method, spray drying method, microfluidic method, and particle aggregation (Thomson et al., 1995; Sinha and Trehan, 2003; Kempen et al., 2006; Jiao et al., 2012; Kemala et al., 2012; Velasco et al., 2012; Dreifke et al., 2013; Huang et al., 2014). Each of these methods has its own unique advantages and is used widely; however, these typical fabrication methods generate microspheres with a broad size distribution because it is difficult to control the microsphere size and shape. Thus, these methods generally produce low yields of microspheres. Furthermore, some of these methods require post-treatment, such as sintering or washing after fabrication, which can damage cells or other components. In some cases (e.g., the spray drying method), it is almost impossible to encapsulate cells in the microsphere due to a harsh and unfavorable environment. In contrast to these conventional methods, electrostatic microencapsulation allows easy control over the size and shape of fabricated microspheres and the inclusion of drugs or proteins in the polymeric matrix (Wu et al., 2010; Bock et al., 2011; Bock et al., 2012). Indeed, the electrostatic microencapsulation method has remarkable advantages that could overcome the drawbacks associated with conventional methods; however, this novel method fabricates microspheres based on polymer. Hence, in the current study, to fabricate homogenous multifunctional hybrid microspheres that contained a drug, ceramic powder, and cells, it was necessary to develop and optimize a method that resulted in the incorporation of all the desired components in the microspheres.

A multifunctional microsphere consists of several components. In this study, the selected components were quercetin (drug), hydroxyapatite (HAp) and alginate (matrix), and MC3T3-E1 cells (cell). Quercetin is a hydrophobic flavonoid phytochemical found in many fruits and vegetables. It has been shown to have anti-oxidative, anti-inflammatory, anti-cancer, anti-aging, angiogenic, and cardiovascular protective effects. It also has positive effects on osteogenesis (D'Andrea, 2015; Li et al., 2015; Mukhopadhyay and Prajapati, 2015; Zhou et al., 2017). However, due to its hydrophobicity, it is challenging to disperse quercetin in aqueous solutions. Thus, an aim of this research was to negate quercetin’s hydrophobic character by attaching it to a carrier. HAp is a one of calcium phosphate (CaP) mineral like as tri-calcium phosphate (TCP) and calcium-deficiency hydroxyapatite (CDHA). It is often used in bone cement because it has the typical CaP family characteristics required for functionality, such as bioactivity, biocompatibility, and osteoconductivity (Nilsson et al., 2002). Thus, HAp was used in this study due to its osteoconductivity and ability to sustainably release drugs—in this case, hydrophobic quercetin. Alginate was selected as the matrix because of its ease of control and crosslinking capacity. It is a natural polymer derived from brown algae commonly used in biomedical applications because of its low toxicity and biocompatibility (Lee and Mooney, 2012). Moreover, these two specific components, HAp and alginate, are well-used components for providing positive effects on bone tissue regeneration due to their complementary interactions. Even for high biocompatibility, alginate scaffolds lack in mechanical strength and have minor applications in bone tissue engineering. Due to the lack of mechanical strength in alginate scaffold for mimicking the function of natural bone, it is combine with inorganic materials, such as HAp, to enhance the strength (Olderøy et al., 2012; Venkatesan et al., 2015). Also, HAp could help and use to enhance cell proliferation and cell growth because of the lack of binding sites of alginate to cells. (Turco et al., 2009; Petrović et al., 2012). To investigate whether cells with osteogenic behavior could be included in the hybrid microspheres, the MC3T3-E1 cell line was selected, which is an osteoblast precursor cell line derived from mouse calvarias.

The aim of this study was to fabricate a multifunctional microsphere, called a hybrid microsphere, based on a natural polymer that contained ceramic powder, cells, and a drug and examine its efficacy as a delivery vehicle and its effects on bone regeneration. To overcome the disadvantages associated with conventional methods, a novel fabrication method was used. The preparation of a homogenous mixture of alginate-HAp nanoparticles containing quercetin was optimized before encapsulation of the scaffold, drug, and cell components into microspheres. Hybrid microsphere size and the associated cell viability and quercetin-releasing behavior at different concentrations were investigated. In addition, the effect of quercetin on cell differentiation was studied.

## Materials and methods

**SCHEME 1:**
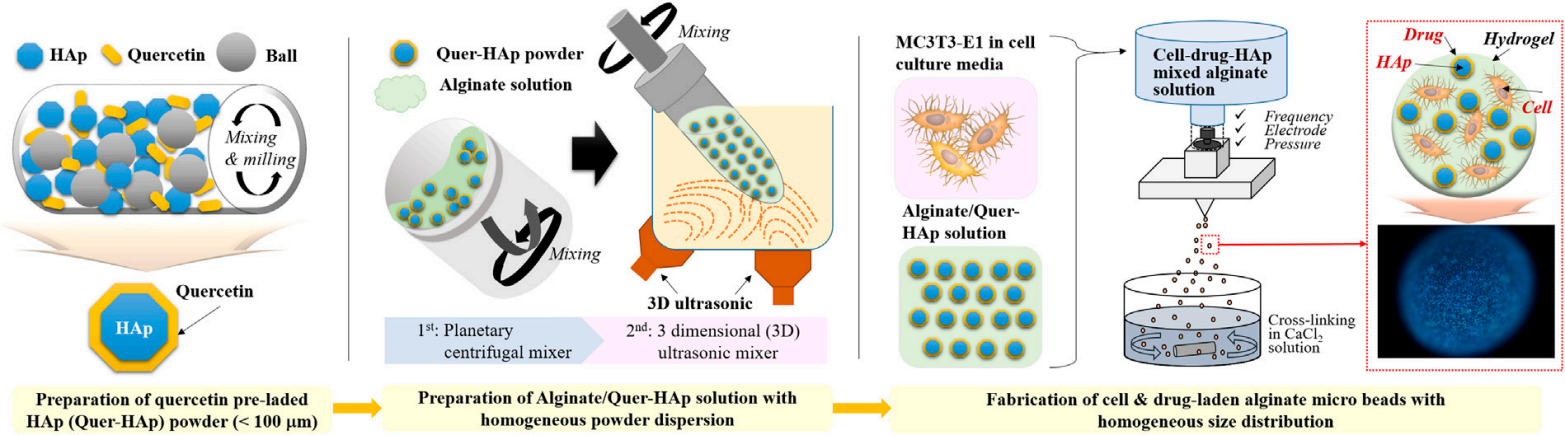
Schematic image of the fabrication of alginate/quercetin-preloaded HAp hybrid microspheres.

The alginate-HAp hybrid microspheres were prepared using the three steps shown in Scheme 1.

### Preparation of alginate/quercetin-preloaded HAp mixture

Quercetin dehydrate (WAKO Chemicals, United States) preloaded HAp (particle size ≤50 nm, CG bio, South Korea) powder was prepared by surface absorption. In a milling jar, 30 g of HAp and 0.24 g of quercetin (8 wt%) were mixed *via* ball milling with 150 ml of ethanol and 200 g of zirconia ball overnight at 200 rpm. Then, the quercetin-preloaded HAp in ethanol was dried for 3 days at room temperature (25°C) to prevent the loss of quercetin. After drying completely, each quercetin-preloaded HAp was filtered through 100 μm sieve. Sodium alginate powder (MW 250,000 mol/g; FMC BioPolymer, United States) was dissolved in distilled water to prepare a 1.25% (w/v) alginate solution. Both HAp and quercetin-preloaded HAp were dispersed in the alginate solution at different organic to inorganic weight ratios (1:0.2, 1:0.5, and 1:1). Two pretreatment steps were necessary to ensure homogenous dispersion of HAp and quercetin-preloaded HAp in the alginate solution. First, a planetary centrifugal mixer (ARE-310, THINKY, United States) was used for 3 min of mixing and 3 min of deforming, both at 2,000 rpm. Second, a 3D ultrasonic mixer (PR-1, THINKY, United States) was used for 15 min at 80 rpm with sonication.

### Determination of HAp powder homogeneity in alginate solution

HAp powder was stained with alizarin red S to determine its dispersion in the alginate solution. HAp powder was dyed directly with 2% alizarin red S and washed with excess water until no red dye was observed while stirring with a rotator. After washing, the stained HAp powder was dried and sieved (100 μm). A 1.25% (w/v) alginate solution was prepared, and the stained HAp powder was prepared at a 1:1 ratio (alginate:HAp). The mixture was treated in various ways: stirring for 6 min, stirring for 21 min, planetary centrifugal mixer for 6 min, planetary centrifugal mixer for 21 min, and planetary centrifugal mixer for 6 min and 3D ultrasonic mixer for 15 min. Immediately after each treatment, homogeneity was determined by observing 1 ml of mixture using optical microscopy (IX71; Olympus, Japan) at ×4 magnification.

### Viscosity measurement

Alginate/HAp mixtures were prepared at ratios of 1:0.2 and 1:1 (alginate:HAp). The mixtures were mixed in different ways, and then the viscosity of a 1.5 ml aliquot was measured using a rheometer (DHR-1, TA instruments, United States) and a flat Peltier plate (40 mm, crosshatched) at 38°C with a shear rate of 0.1–1.0 (1/s) and flow sweep conditions. The viscosity of alginate/0 wt% and 8 wt% quercetin-preloaded HAp mixture was measured under the same conditions.

### Cell culture

The mouse calvaria-derived preosteoblast cell line MC3T3-E1 subclone 14 (American Type Culture Collection, United States) was cultured in alpha-minimum essential medium (α-MEM, Gibco, United States) with 10% fetal bovine serum (Gibco, United States) and 1% penicillin streptomycin (Gibco, United States) at 37°C with a 5% CO_2_ atmosphere. Cultured media was refreshed every 2–3 days. After rinsing the cells once with phosphate buffered saline (PBS), the cells were harvested by treatment with 0.25% trypsin-ethylenediaminetetraacetic acid (EDTA; 4 min), followed by centrifugation (3 min at 1,500 rpm).

### Fabrication of alginate/quercetin-preloaded HAp hybrid microspheres

The harvested cells, at 5 × 10^6^ cells/ml, were added to the homogenous alginate/0 and 8 wt% quercetin-preloaded HAp mixtures. The hybrid microspheres were fabricated using an electrostatic microencapsulator (B-390, BUCHI Corporation, United States) with the following conditions: frequency of 1,800 Hz, electrode with 600 V, and pressure of 400–450 mbar. After all the solution formed hybrid microspheres, the cell-laden alginate/quercetin-preloaded HAp hybrid microspheres were crosslinked in 100 mM CaCl2 for 30 min with stirring. The hybrid microspheres were then transferred to 6-well plates for culturing in cell-culture medium.

To study the osteogenic differentiation of the cells in the hybrid microspheres, cell-culture medium was replaced with osteogenic-differentiation medium, which consisted of culture medium plus 50 μg/ml L-ascorbic acid 2-phosphate sesquimagnesium salt hydrate (Tokyo Chemical Industry Co., Japan) and 10 mM β-glycerophosphate disodium salt, pentahydrate (EMD Millipore, United States).

### Hybrid microsphere size analysis

Optical images of the alginate/quercetin-preloaded hybrid microspheres were obtained by optical microscopy at ×4 magnification. The size distribution and aspect ratio of each type of hybrid microsphere were measured using ImageJ (n = 200).

### Analysis of drug loading capacity and release

An equal concentration (8 mg/ml) of samples for each group (free quercetin, quercetin-preloaded HAp, pure HAp for control) was prepared. Every group was dissolved by vortexing in ratio of 50:50 dimethyl sulfoxide/hydrochloric acid (DMSO/HCl). After all groups were dissolved completely in DMSO/HCl buffer, the optical density of each 100 μl sample was measured using a spectrophotometer (spectramax M2; Molecular Devices, United States) at a wavelength of 375 nm. The drug loading capacity was calculated using the equations:

$$\text{Loading efficiency}\left(\text{\%}\right)=\frac{\text{Amount of loaded quercetin on surface of HAp}}{\text{Total amount of quercetin}}\times 100$$

The alginate/0 wt% and 8 wt% quercetin-preloaded HAp hybrid microspheres without cells, the microspheres containing only quercetin, and the microspheres containing unlinked quercetin and HAp were prepared for the drug release test at a ratio of 1:0.5 (alginate: quercetin-preloaded HAp). Each group of hybrid microspheres was submerged in 1 ml of Hanks’ Balanced Salt solution (HBSS) with 0.02% sodium azide and kept at 37°C under static conditions. Sampling was done at each time point, and the removed liquid was replaced with buffer. The optical density of each 200-μl sample was measured using a spectrophotometer at a wavelength of 375 nm.

### Cell viability

Cell viability was assessed using a modified protocol of the Invitrogen live/dead viability/cytotoxicity kit for mammalian cells (Invitrogen, United States). Cells were assayed at two time points: immediately after crosslinking and after 3 weeks of culturing. Cells were stained with the fluorescent calcein-AM and EthD-III, and Hoechst 33,342 dye (Sigma, United States). The fluorescent images were obtained by confocal microscopy (TCS SP8 X, Leica, Germany). Flow cytometry (Novocyte, ACEA Bioscience, United States) was used to quantitate the living and dead cells at 3 weeks. Cells were treated with EDTA (37°C for 30 min) before staining with a live/dead fixable staining kit (Promo cell GmbH, Germany) according to the product instructions.

### Quantitative analysis of alkaline phosphatase activity and calcium formation

The cell-laden alginate/quercetin-preloaded HAp hybrid microspheres were continuously cultured for 3 weeks. At predetermined time points (1, 2, and 3 weeks), 1 ml of hybrid microspheres was transferred to a test tube. Cell lysis buffer (1 ml) was added to each tube, and the hybrid microspheres were broken apart using a homogenizer. To determine alkaline phosphatase (ALP) activity, p-nitrophenyl phosphate (pNPP) was added as the ALP substrate, and optical density was measured at 405 nm using a spectrophotometer. A commercial calcium assay kit (Pointe scientific. INC, United States) was used for the quantitative determination of calcium ions, with optical density measured at 570 nm using a spectrophotometer.

### Osteogenic marker gene expression

After 3 weeks of culture in osteogenic differentiation medium, reverse transcription (RT-PCR) and quantitative real-time polymerase chain reaction (qPCR) analyses were performed to evaluate the expression of relevant osteogenic markers. To extract RNA and reverse transcribe it into complementary DNA (cDNA), the RNAiso extraction reagent (Takara, Japan) was used, according to the product manual. As per the instructions, 20 µl of solution was incubated in a PCR thermal cycle (C1000; Bio-Rad, United States), initially at 25°C for 5 min, then at 45°C for 1 h, and finally at 95°C for 5 min. Gene expression was quantitatively determined using real-time PCR equipment (TP900, Takara, Japan) and TB Green Fast qPCR Mix (Takara, Japan). The cycling conditions were as follows: initial denaturation cycle at 95°C for 30 s, 40 cycles at 95°C degeneration for 5 s, and 60°C of annealing and extension for 10 s. The cycle threshold for every transcript expression was exported from Takara dice real-time software. The comparative 2^(-△△Ct) method was used to calculate the relative abundance of mRNA. Primers used included: 5′-ATG​TCC​CAG​CAG​GAT​TTG​AG-3' (sense) and 5′-CCT​AAT​GCT​TTT​TCT​GC-3' (anti-sense) for collagen type I, 5′-GCC​TTT​GAG​GTT​TTT​GGT​CA-3' (sense) and 5′-AAC​CCA​GAC​ACA​AGC​ATT​CC-3' (anti-sense) for ALP, 5′-GTT​TGG​CTT​TAG​GGC​AGC​AC-3' (sense) and 5′-GGA​GGG​TGC​AGA​ACA​GAC​AA-3' (anti-sense) for osteocalcin, and 5′-CCA​TCC​AAT​CGG​TAG​TAG​CG-3' (sense) and 5′-GTA​ACC​CGT​TGA​ACC​CCA​TT-3' (anti-sense) for 18S ribosomal RNA.

### Statistics

Data of alkaline phosphatase activity, calcium formation and quantitative PCR were analyzed by one-way analysis of variance (ANOVA), with Tukey simultaneous tests used to identify differences between individual groups of hybrid microspheres, using Origin software. Each cell activity experiments were repeated at least three times with 4 different samples in each group, and *p* < 0.05 was considered statistically significant.

## Results

### Control of HAp particle dispersion in alginate solution

**FIGURE 1:**
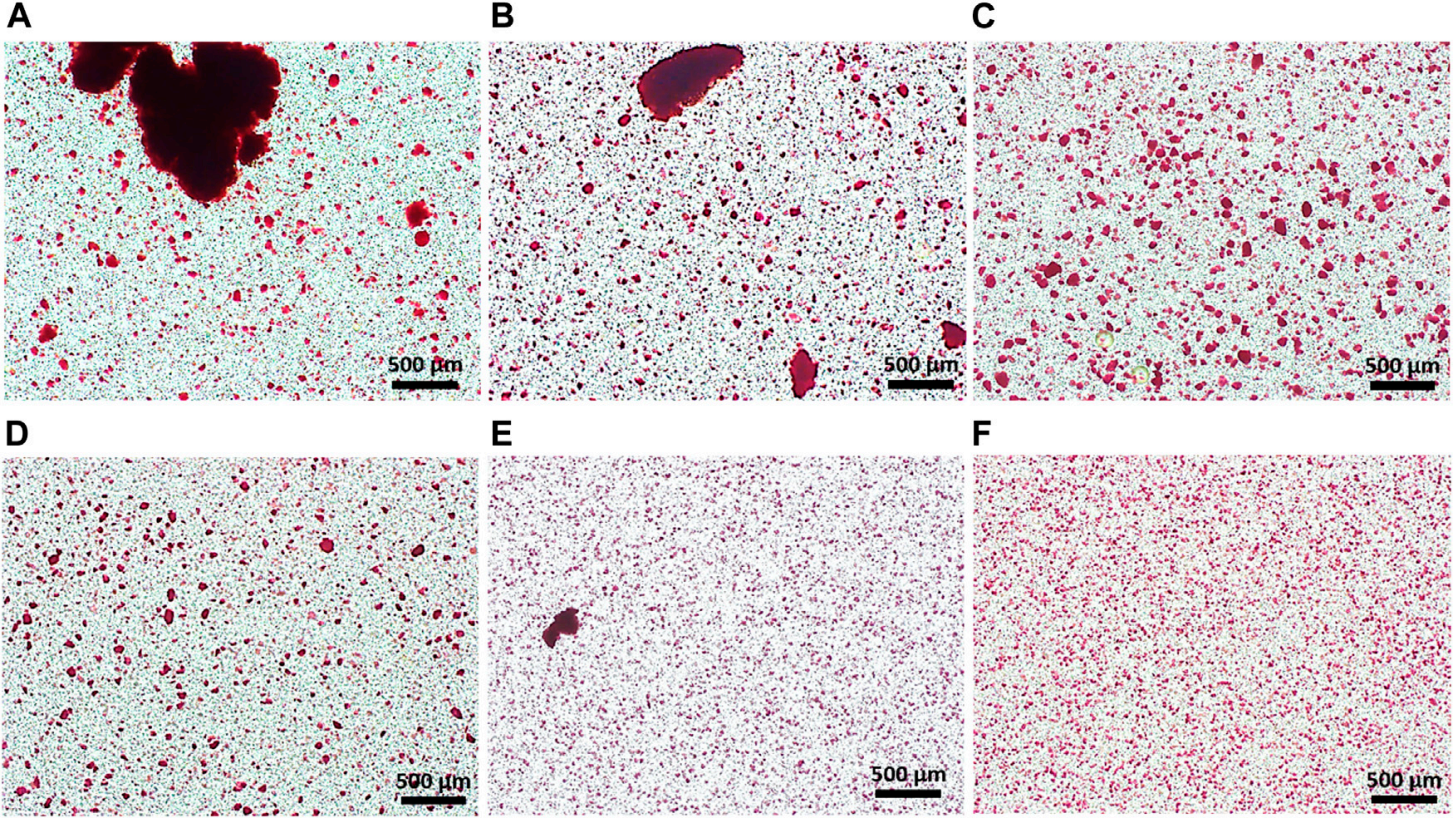
Homogeneity of HAp powder in alginate solution; stirring for **(A)** 6 min and **(B)** 21 min; planetary centrifugal mixing for **(C)** 6 min and **(D)** 21 min; 3D ultrasonic mixing for **(E)** 15 min **(F)** planetary centrifugal mixing for 6 min and 3D ultrasonic mixing for 15 min.

HAp nanoparticles should be homogeneously dispersed in alginate solution to prevent aggregates from clogging the spraying nozzle and to achieve continuous production of microspheres. However, it is difficult to make HAp nanoparticles disperse homogeneously in alginate solution because the alginate solution is viscous and the nanoparticles have a tendency to adhere to each other. We therefore needed to optimize the preparation of the alginate/HAp mixture. Various pretreatments were tested for their effect on the homogeneity of the HAp-alginate mixture. Conventional stirring with a magnetic bar, the use of a planetary centrifugal mixer, and/or the use of a 3D ultrasonic mixer for different times were applied. The HAp powder was pre-stained with alizarin red S to track it in the final mixtures. The conventional stirring treatment by magnetic stirrer for 6 and 21 min resulted in the lowest homogeneity (Figures 1A,B). A large amount of agglomeration of HAp powder in alginate solution was detected. Pretreatment using a planetary centrifugal mixer for 6 and 21 min provided better homogeneity compared to the stirring treatment, but aggregations were still found (Figures 1C,D). Compared to conventional stirring by magnetic bar and planetary centrifugal mixer, HAp nanoparticles were well dispersed in the alginate solution when mixed by a 3D ultrasonic mixer; however, there were a few aggregations detected (Figure 1E), and the nozzle was clogged during fabrication. To further enhance the homogeneity, a two-step mixing process was applied, which involved both the planetary centrifugal mixer and the 3D ultrasonic mixer. With this two-step treatment of planetary centrifugal mixing for 6 min and 3D ultrasonic mixing for 15 min, the HAp powder was sufficiently dispersed in the alginate solution and the highest homogeneity was recorded (Figure 1F). The resultant mixture was of sufficient quality to be used for fabricating hybrid microspheres.

### Preparation of hybrid microspheres using electrostatic microencapsulation

**FIGURE 2:**
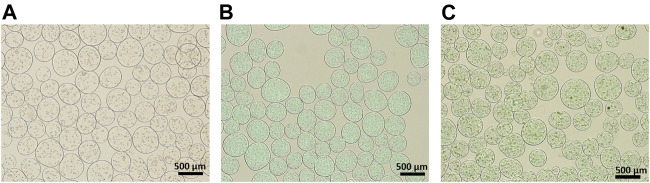
Optical images of the 1:1 ratio of cell-encapsulated alginate/different composite hybrid microspheres. **(A)** Alginate with cell (without HAp nor quercetin) **(B)** alginate:HAp; and **(C)** alginate:8 wt% quercetin-preloaded HAp.

**FIGURE 3:**
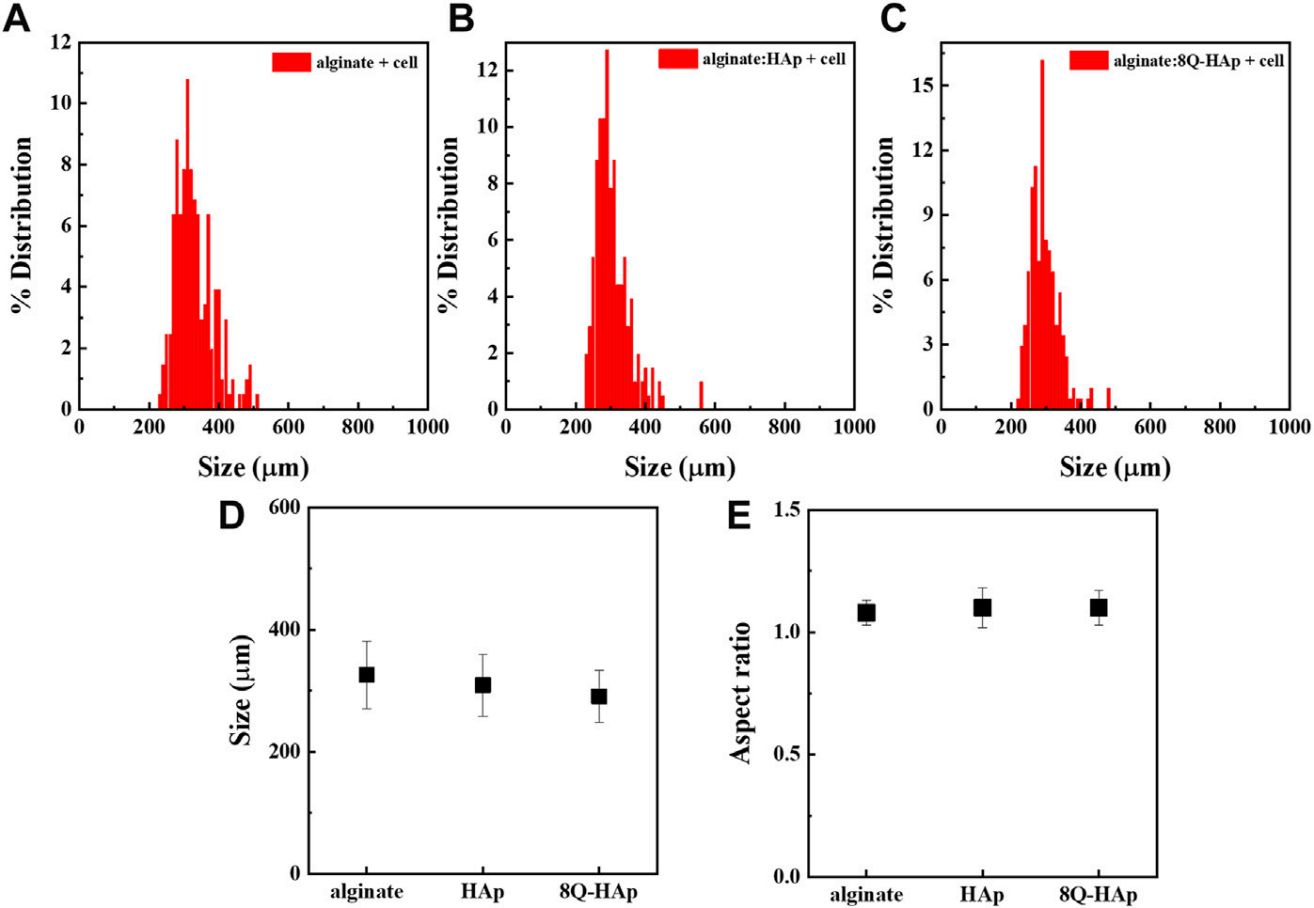
Size distribution of different concentrations of cell-encapsulated quercetin-preloaded HAp hybrid microspheres. **(A)** Alginate with cells (without HAp nor quercetin) **(B)** alginate:HAp **(C)** alginate: 8 wt% quercetin-preloaded HAp **(D)** size analysis for each group; and **(E)** aspect ratio for each group.

Hybrid microspheres were fabricated using electrostatic microencapsulation under optimized conditions for frequency, electrode, and pressure, which largely influence both the size and shape of microspheres. Microsphere shape was affected by the frequency and electrode, and the pressure simply affected microsphere size. We optimized the conditions for fabricating spherical and identical-sized hybrid microspheres (1,800 Hz frequency, 600 V electrode, and 450 mbar pressure). The effect of the addition of HAp nanoparticles and quercetin-preloaded HAp nanoparticles on microsphere formation was examined (Figure 2), and it was found that the microsphere size decreased with the addition of HAp and the drug, but the change was almost negligible. That is, the size of microspheres with cells only was 325.8 ± 55.2 μm, whereas the size of microspheres with HAp and cells was 309.1 ± 51.0 μm. The size of microspheres with 8 wt% quercetin-preloaded HAp and cells was 290.7 ± 42.5 μm. The aspect ratio of each group was measured and found to be almost 1 for all types, indicating that all microspheres had good spherical morphology (Figure 2 and Figure 3E ). The alginate microspheres with cells showed a narrow size distribution. Furthermore, we achieved a similarly good size distribution of microspheres after the addition of HAp nanoparticles and HAp nanoparticles containing the drug. That is, we fabricated alginate/HAp hybrid microspheres with cells (MC3T3-E1) and a drug (quercetin) using electrostatic microencapsulation with superior yield.

### Release of quercetin from hybrid microspheres

**FIGURE 4:**
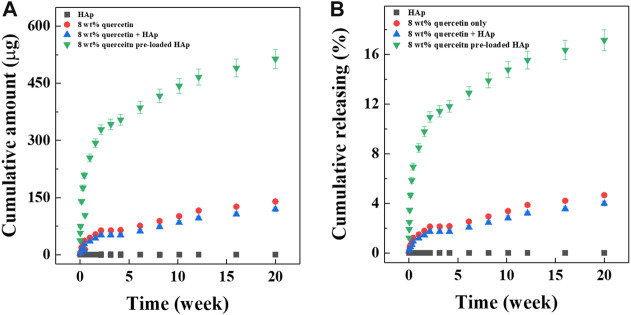
Drug releasing test. **(A)** Cumulative release of alginate:8 wt% quercetin-preloaded HAp microspheres and **(B)** cumulative release percentage of alginate:8 wt% quercetin-preloaded HAp hybrid microspheres.

The data demonstrating the release of quercetin from the hybrid microspheres are shown in Figure 4. To determine the effect of preloading quercetin onto the HAp on drug release performance, four types of microspheres were prepared with same amount of quercetin considered 10.68% of loading efficiency, in different way of encapsulated: alginate/quercetin microspheres, alginate/HAp microspheres, alginate/quercetin/HAp microspheres with simple mixing, and alginate/quercetin-preloaded HAp microspheres. The accumulative amounts of quercetin released and the cumulative percentages of quercetin released over time are shown in Figures 4A,B. Released quercetin from the two microsphere types that included free (unbound) quercetin was hardly detected because of its extremely low solubility in aqueous solutions. Only 4% (accumulative percentage) of the total amount of quercetin was detected in these two groups. Meanwhile, considerably higher quantities of released quercetin were detected in the samples of hybrid microspheres that contained quercetin-preloaded HAp. The hybrid microspheres with 8 wt% quercetin-preloaded HAp showed an impressive release of 513.9 ± 25.2 μg over the 20 weeks, indicating the effectiveness of preloading the drug onto HAp nanoparticles on increasing quercetin solubility. This tendency was reflected in the cumulative releasing percentage (Figure 4B). During the 20-weeks test period, the hybrid microspheres that contained 8 wt% quercetin-preloaded HAp released 17% of the total amount of quercetin.

### Cell viability

**FIGURE 5:**
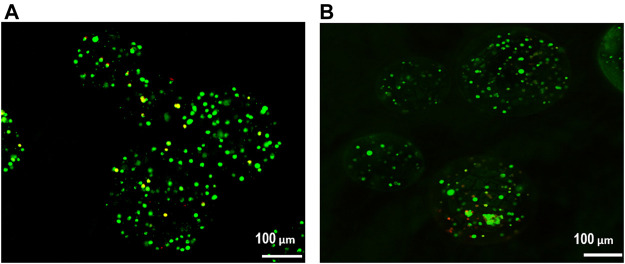
Cell viability was assessed using a live/dead assay. **(A)** After crosslinking and **(B)** after 3 weeks of culture in osteogenic differentiation medium.

**FIGURE 6:**
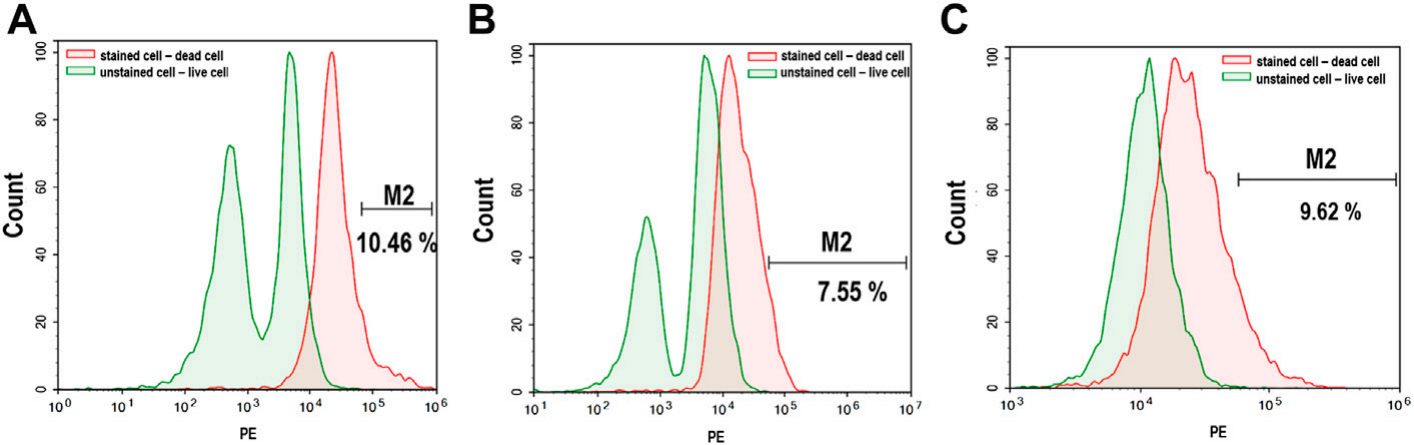
Live/dead cell signaling via flow cytometry after 3 weeks of culture. **(A)** Alginate **(B)** alginate: HAp; and **(C)** alginate:8 wt% quercetin-preloaded HAp.

The effect of alginate crosslinking in CaCl_2_ on cell viability was determined. The green signals indicate live cells, and the red signals indicate dead cells. Cell viability was assessed immediately after crosslinking and after 3 weeks of culturing in osteogenic differentiation medium. As shown in Figure 5, there was no significant critical damage to cells during fabrication or culture, evidenced by the numerous green-stained (live) cells compared to the few red-stained (dead) cells. Flow cytometry analysis confirmed high cell viability. As shown in Figure 6, nearly 90% of the cells were still alive in every group of hybrid microspheres after 3 weeks in culture. Specifically, 89.5% were viable in the alginate microspheres, 92.5% in the microspheres containing HAp, and 90.4% in the hybrid microspheres containing 8 wt% quercetin-preloaded HAp.

### Osteogenic differentiation behavior of cells in hybrid microspheres

**FIGURE 7:**
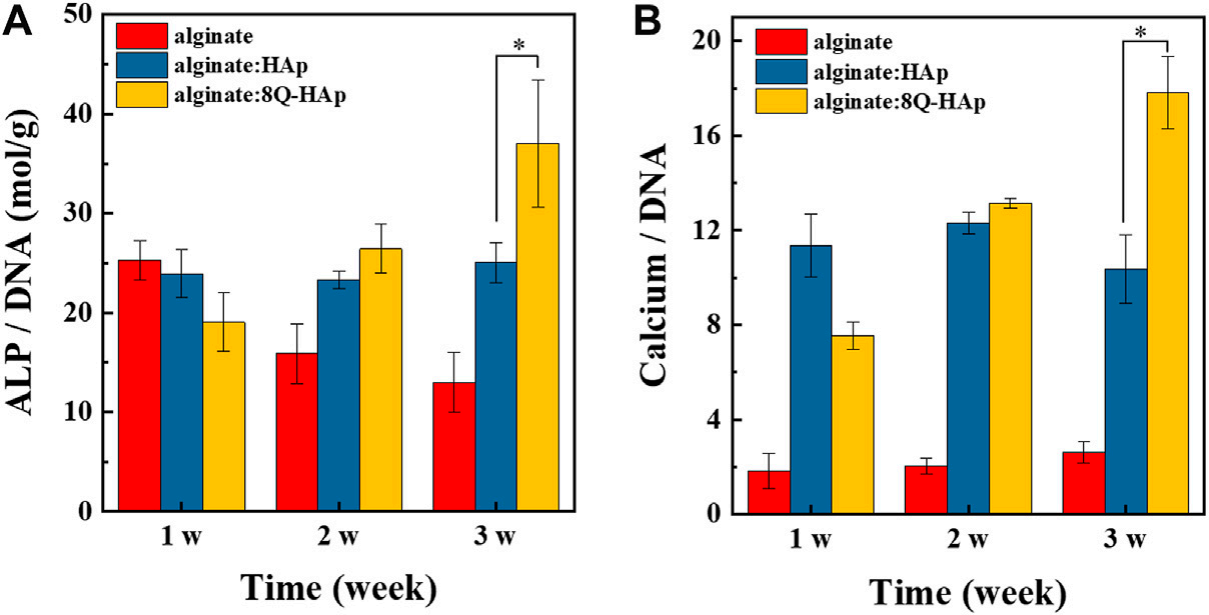
*In vitro* osteogenic differentiation in each group of 1:1 (alginate: quercetin-preloaded HAp) hybrid microspheres. **(A)** ALP/DNA and **(B)** calcium/DNA. (n = 4), *p < 0.05.

After 3 weeks in culture, the effect of quercetin on the osteogenic differentiation behavior of cells in each group of hybrid microspheres was assessed. ALP and calcium ion levels were detected, and the results are shown in Figure 7. From week 2, the cells in the hybrid microspheres containing the drug showed higher quantities of ALP (8 wt% quercetin-preloaded HAp: 27.4 ± 1.2 mol/g ALP/DNA) than those in the hybrid microspheres without the drug and without HAp (without HAp and quercetin: 14.6 ± 1.2 mol/g, without quercetin: 25.9 ± 0.9 mol/g ALP/DNA). Subsequently, there were larger and significant differences at week 3: cells in microspheres with 8 wt% quercetin-preloaded HAp had 38.9 ± 5.6 mol/g ALP/DNA; cells in microspheres without HAp and quercetin had 14.2 ± 1.5 mol/g; and cells in microspheres without quercetin had 25.9 ± 1.1 mol/g. These findings indicate that quercetin affects cell differentiation, with the highest amount of ALP detected in cells in hybrid microspheres containing quercetin.

In terms of calcium ion accumulation, the cells in the hybrid microspheres that contained 8 wt% quercetin-preloaded HAp showed the highest quantity of calcium ions (17.8 ± 1.5 mg/mg calcium ion/DNA) after 3 weeks of culturing (microspheres without HAp and quercetin: 2.6 ± 0.5 mg/mg calcium ion/DNA, microspheres without quercetin: 10.4 ± 1.5 mg/mg calcium ion/DNA). Similar to the ALP quantity results, the hybrid microspheres containing quercetin-preloaded HAp showed the best bone remodeling effects on cells.

### Osteogenic marker gene expression

**FIGURE 8:**
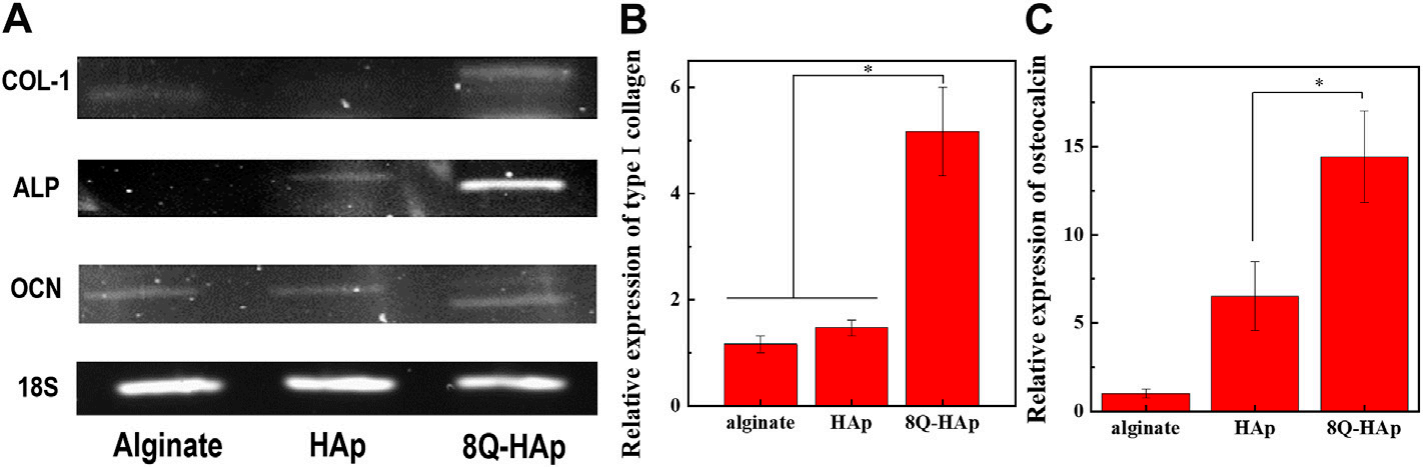
Osteogenic marker gene expression at week 3. **(A)** RT-PCR analysis of the expression of an osteogenic specific gene and **(B–C)** qRT-PCR results for genes monitored in each group (n = 3), *p < 0.05: COL-1 **(B)** and osteocalcin **(C)**.

Gene expression analysis was carried out by RT-PCR and qPCR to assess the osteogenic activation of the MC3T3-E1 cells encapsulated in the hybrid microspheres. RT-PCR analysis indicated production of COL-1, ALP, and osteocalcin (OCN) in cells after 3 weeks in culture with osteogenic differentiation medium. Regarding the expression of COL-1, the cells in the hybrid microspheres containing 8 wt% quercetin-preloaded HAp demonstrated a slightly higher level than the other two groups: cells in hybrid microspheres containing HAp without quercetin and cells in microspheres with cells only. In terms of ALP expression, the cells in hybrid microspheres containing quercetin-preloaded HAp showed a significantly higher level compared to cells in other types of hybrid microspheres. Regarding OCN expression, the cells in every type of hybrid microsphere showed comparable levels of expression (Figure 8A).

The qPCR results were used to analyze the relative gene expressions of COL-1 and OCN after 3 weeks in culture (Figures 8B,C). The expression of the differentiation genes COL-1 and OCN was significantly different across the groups. COL-1 expression was 5.2-fold higher in cells in the hybrid microspheres containing 8 wt% quercetin-preloaded HAp than in cells in microspheres containing cells only. In addition, the expression was 1.5-fold higher in cells in the microspheres without quercetin than in cells in microspheres containing cells only. The differences were more marked in OCN expression. The hybrid microspheres containing 8 wt% quercetin-preloaded HAp induced 14.4-fold greater expression than the microspheres without quercetin and HAp. The microspheres without quercetin induced 6.5-fold greater expression than the microspheres containing cells only.

## Discussion

Microspheres are effective drug delivery vehicles for the sustained release of target biomolecules. They are also considered a useful bone substitute and are applied in complex or irregular defect sites during orthopedic, maxillofacial, and dental surgery to induce bone tissue regeneration (Jyothi et al., 2010; Huang et al., 2014; Ingavle et al., 2019). Polymers are typically used for the former application, whereas ceramics are used for the latter. There are several conventional techniques for the fabrication of microspheres, such as spray drying, microfluidic, and w/o emulsion processes based on polymers, ceramics, or their composites (Dreifke et al., 2013; Huang et al., 2014; Gupta V. et al., 2017; Raja et al., 2021). Even though these fabrication methods are well-known, they have certain technical limitations, such as difficulties producing high yields of specific sized products, restrictions on applicable materials, and challenges in creating multifunctional products (Jiao et al., 2012; Kemala et al., 2012). Spray drying is used to produce both polymer and ceramic microspheres but not to incorporate cells. Both microfluidic and w/o emulsion processes are typically used to produce polymer microspheres for drug or cell delivery, but they are not amenable to ceramics.

Fortunately, the microencapsulation process is a new option for fabricating microspheres with good size distribution, high production efficiency (Bock et al., 2011; Bock et al., 2012), and a useful carrier function for drugs, and cell. The size and shape of the microspheres can be controlled by adjusting the nozzle size, frequency, electrode, and pressure. Until now, microencapsulation has not been considered as a means for producing polymer-ceramic hybrid materials or ceramics because it is extremely difficult to use these materials in this system due to the micro-sized nozzle. A viscous fluid, such as alginate, gelatin, and poly (lactic-co-glycolic acid) (Singh et al., 2010; Corstens et al., 2019), is generally required as a basic material for electrostatic microencapsulation. However, it is challenging to prevent the aggregation of the ceramic nanoparticles in a viscous polymer. Such aggregates can clog the nozzle and consequently cause the microsphere fabrication to fail.

Here, we report a solution for the preparation of multifunctional polymer-ceramic hybrid microspheres with drug- and cell-carrier activity using microencapsulation (Scheme 1). First, we optimized the raw material preparation process to enable hybrid microspheres to incorporate and deliver the hydrophobic drug quercetin. We coated HAp with quercetin through ball-milling in ethanol to prevent quercetin conglomeration, which can reduce its solubility in aqueous conditions, and to accordingly increase quercetin solubility and efficiency. Second, we prepared an alginate solution with a homogeneous distribution of quercetin-preloaded HAp through the use of a combination of 3D ultrasonic treatment and planetary centrifugal mixing. The 3D ultrasonic treatment was essential to prevent the aggregation of HAp in the viscous alginate solution (Figure 1). Without this treatment, the HAp aggregated (Figures 1A–D) and caused nozzle clogging. Spherical hybrid microspheres were successfully fabricated with good reproducibility and homogeneous size distribution. We obtained a good production yield compared to conventional fabrication methods because additional sorting steps were not required. (Pham et al., 2002; Jiao et al., 2012; Kemala et al., 2012) (Figure 2 and Figure 3).

As a third step, cells were added to the mixture. They were not added before because the earlier sonication treatment might have damaged the cells. The target size of the hybrid microspheres containing cells was 100–400 μm in diameter to ensure that the cells were within the limits of oxygen diffusion (Leong and Wang, 2015). While the fabrication of cell-laden hybrid microspheres must be under conditions that are favorable to cell viability, the conditions must also allow the regulation of the size and shape of the hybrid microspheres. Frequency and electrode affected the shape of the hybrid microsphere, and pressure mostly affected their size. Of all the factors, frequency was the most important because the cells could be directly damaged by high frequency. For that reason, the frequency had to be relatively low for optimization (Velasco et al., 2012; Haiyi et al., 2019).

The hybrid microspheres that contained cells and quercetin-preloaded HAp were spherical, with an aspect ratio of 1 (Figure 3E). Despite their different components, each type of hybrid microsphere was within the target size range, with diameters of 290–330 μm.

Cell viability was a factor of concern during hybrid microsphere fabrication, because high cell viability was necessary to verify the effects of quercetin on the cells in the hybrid microspheres. Therefore, the quantities of living and dead cells were analyzed immediately after fabrication and after 3 weeks in culture (Figure 6). The results showed that the fabrication and culture conditions were cell-friendly, and thus demonstrated that it might be feasible to deliver not only cells but also bioactive molecules, such as proteins and aptamers.

The cell laden hybrid microsphere was obviously contained drugs concurrently during fabrication. As long as, quercetin has been claimed to have beneficial biological effects such as osteogenesis, angiogenesis, but with the low-water solubility limited its applications (Hausman et al., 2006; Zhou and Lin, 2014; Gupta S. K. et al., 2017; Zhou et al., 2017).

To overcome the hydrophobic nature of quercetin, it was attached to HAp, creating a physical drug-ceramic interaction between quercetin and HAp (Diaz-Rodriguez et al., 2018). The cumulative amount and percentage of quercetin released from each type of hybrid microsphere is shown in Figure 4. Each hybrid microsphere type exhibited different release behaviors. Due to the hydrophobicity of quercetin, the hybrid microspheres that contained quercetin only or quercetin and HAp separately released the drug extremely slowly. It was assumed that when quercetin was encapsulated in the water-based hybrid microspheres, a hydrophobic interaction occurred between the drug molecules and caused limited release (Gu et al., 2017). However, the hybrid microsphere containing 8 wt% quercetin-preloaded HAp showed sustainable release, indicating that the drug-drug hydrophobic interaction is a relatively stronger interaction than the drug-ceramic interaction (Diaz-Rodriguez et al., 2018). This leads to the possibility that the relatively weak drug-ceramic interaction could be broken more easily than the drug-drug interaction, which could facilitate the release of quercetin. As a result, quercetin preloaded on the HAp surface was released in a sustained manner over the long term, and the quantity released was dependent on the concentration of loaded quercetin.

Regarding the differentiation activity of the cells encapsulated in the hybrid microspheres in this study, the differentiation of the MC3T3-E1 cells was influenced by quercetin concentration and the presence of HAp. ALP was detected to monitor cell differentiation, as it is expressed in the early stages of osteogenesis and hydrolyzes organic phosphates, which causes the release of phosphorous ions and extracellular matrix mineralization (Bellows et al., 1993). Calcium ion levels were also detected to monitor the amount of mineralization that occurred within the hybrid microspheres. As shown in Figure 7, the hybrid microspheres containing quercetin-preloaded HAp induced the highest ALP and calcium ion levels. In addition, hybrid microspheres without quercetin-loaded HAp induced higher amounts of ALP and calcium ions than those that contained cells only (without HAp or quercetin).

The osteogenic activity of the cells was further assessed by the evaluation of COL-1, ALP, and OCN gene expression. COL-1 is a major constituent of the extracellular matrix of bone tissue and has been found at high levels during the early stage of osteogenesis. In turn, OCN is regarded as a late maker of osteogenesis with a high level of expression associated with the maturation of osteoblasts. (Christenson, 1997). In the RT-PCR results, the cells in the hybrid microspheres containing 8 wt% quercetin-preloaded HAp showed the highest gene expression for every marker. The differences were the most significant for COL-1 and ALP gene expression. Similarly, the qPCR results showed that the cells in the hybrid microspheres containing 8 wt% quercetin-preloaded HAp had the highest gene expression for COL-1 and OCN. Notably, the cells in the hybrid microspheres without quercetin also showed a slightly higher level of COL-1 and a significantly higher level of OCN expression compared to the microspheres without HAp or quercetin. These findings show that even HAp affected cell differentiation directly (Nagahara et al., 1992). Thus, HAp and quercetin-preloaded HAp both demonstrated effects on the bone remodeling activity of cells.

In this study, the hybrid microsphere fabrication process was optimized, and it was possible to verify the effects of the internal components of the hybrid microspheres. This indicates that these microspheres could be developed as multifunctional delivery vehicles of cell and drug. When these cell and drug delivered simultaneously to target tissue, it could be providing effective biomolecule to accelerate differentiation in that target site better than individual delivery system. Thus, this study demonstrates the possibility of producing multifunctional carriers that deliver drugs and cells that aid bone regeneration.

## Conclusion

Cell-laden hybrid microspheres containing quercetin and HAp were successfully fabricated using a novel process with high productivity. The process involved preparing a mixture with homogenously dispersed HAp and drug through a two-step pretreatment. The size of the alginate/HAp hybrid microspheres was regulated, and they ranged from 290 to 330 μm and had a spherical shape with an aspect ratio of 1. The high cell viability proved that the fabrication and culture conditions were suitable for the cells. The microspheres were found to release the hydrophobic drug that was physically adsorbed to the ceramic powder, and the release was sustained over the long term. Moreover, the cell-laden hybrid microspheres containing quercetin showed potential as an effective bone substitute over 3 weeks of culturing; they enhanced the cells’ osteogenic differentiation. Hence, hybrid microspheres that contain cells, a drug, and ceramic powder have great potential as drug and cell delivery vehicles in the bone tissue regeneration field.

## Data Availability

The original contributions presented in the study are included in the article/supplementary materials, further inquiries can be directed to the corresponding authors.
